# The Establishment of a Noninvasive Bioluminescence-Specific Viral Encephalitis Model by Pseudorabies Virus-Infected NF-κBp-Luciferase Mice

**DOI:** 10.3390/vetsci9030113

**Published:** 2022-03-03

**Authors:** Hui-Wen Lin, Meilin Wang, Pei-Jane Tsai, Yi-Ju Lee, Ming-Chang Hsieh, Dah-Yuu Lu, Wei-Li Hsu, Ming-Shiou Jan, Yuan-Yen Chang

**Affiliations:** 1 Department of Optometry, Asia University, Taichung 40354, Taiwan; d9138001@asia.edu.tw; 2 Department of Medical Research, China Medical University Hospital, China Medical University, Taichung 40201, Taiwan; 3 Department of Microbiology and Immunology, School of Medicine, Chung Shan Medical University, Taichung 40201, Taiwan; wml@csmu.edu.tw; 4 Clinical Laboratory, Chung Shan Medical University Hospital, Taichung 40201, Taiwan; 5 Department of Medical Laboratory Science and Biotechnology, National Cheng Kung University, Tainan 70101, Taiwan; peijtsai@mail.ncku.edu.tw; 6 Department of Pathology, School of Medicine, Chung Shan Medical University, Taichung 40201, Taiwan; jasmine.lyl@gmail.com; 7 Department of Pathology, Chung-Shan Medical University Hospital, Taichung 40201, Taiwan; 8 Department of Medical Laboratory and Biotechnology, Chung Shan Medical University, Clinical Laboratory, Chung Shan Medical University Hospital, Taichung 40201, Taiwan; cshb183@csh.org.tw; 9 Department of Pharmacology, School of Medicine, China Medical University, Taichung 40201, Taiwan; dahyuu@mail.cmu.edu.tw; 10 Department of Photonics and Communication Engineering, Asia University, Taichung 40201, Taiwan; 11 Graduate Institute of Microbiology and Public Health, College of Veterinary Medicine, National Chung Hsing University, Taichung 40201, Taiwan; wlhsu@dragon.nchu.edu.tw; 12 Institute of Medicine, Chung Shan Medical University, Taichung 40201, Taiwan; 13 Division of Allergy, Immunology and Rheumatology, Department of Internal Medicine, Chung Shan, Medical University Hospital, Taichung 40201, Taiwan

**Keywords:** pseudorabies virus (PRV), nuclear factor-kappa B promoter (NF-κBp)-luciferase mice, proinflammatory mediators

## Abstract

Encephalitis is a rare brain inflammation that is most commonly caused by a viral infection. In this study, we first use an in vivo imaging system (IVIS) to determine whether NF-κBp-luciferase expression could be detected in the brain of pseudorabies virus (PRV)-infected NF-κBp-luciferase mice and to evaluate proinflammatory mediators in a well-described mouse model of PRV encephalitis. In in vitro studies, we used murine microglia (BV-2) cells to demonstrate the PRV-induced encephalitis model entailing the activation of microglia cells. The results indicate that PRV-induced neuroinflammation responses through the induction of IL-6, TNF-α, COX-2, and iNOS expression occurred via the regulation of NF-κB expression in BV-2 cells. In in vivo studies, compared with MOCK controls, the mice infected with neurovirulent PRV exhibited significantly elevated NF-κB transcription factor activity and luciferase protein expression only in the brain by IVIS. Mild focal necrosis was also observed in the brain. Further examination revealed biomarkers of inflammation, including inducible cyclooxygenase (COX)-2, inducible nitric oxide synthase (iNOS), and tumor necrosis factor (TNF)-α and interleukin (IL)-6, both of which constituted proinflammatory cytokines. PRV infection stimulated inflammation and COX-2 and iNOS expression of IL-6 and TNF-α. The presented results herein suggest that PRV induces iNOS and COX-2 expression in the brain of NF-κBp–luciferase mice via NF-κB activation. In conclusion, we used NF-κBp-luciferase mice to establish a specific virus-induced encephalitis model via PRV intranasal infection. In the future, this in vivo model will provide potential targets for the development of new therapeutic strategies focusing on NF-κB inflammatory biomarkers and the development of drugs for viral inflammatory diseases.

## 1. Introduction

In herpesvirus studies, the pseudorabies virus (PRV)—a porcine herpesvirus belonging to the subfamily Alphaherpesvirinae—is an ideal model organism [1]. PRV infects various hosts, including most mammals, other than higher-order primates. Wild PRV infections primarily result in reproductive and respiratory diseases, with low mortality rates in adult pigs, the natural PRV host. In contrast, the affected unnatural host experiences neurological symptoms and eventually leads to death. Given its exceptional tendency to attack synaptically connected neurons, PRV has been investigated as a “live” marker of neuronal pathways [1].

Among alphaherpesvirus, herpes simplex virus (HSV) type 1 is the most characterized [2]. Sporadic acute encephalitis—most commonly caused by HSV—when not treated, has a mortality rate exceeding 70%; even after the administration of antiviral therapy, the rates of neurological deficits and mortality are extremely high. By contrast, PRV infections in susceptible non-porcine species trigger uncontrolled inflammations and eventually fatal encephalitis [3,4]. Our recent studies have shown that PRV-infected macrophages induce inflammatory responses engendering NO and proinflammatory chemokine production, probably through the nuclear factor-kappa B promoter (NF-κBp) pathway [5,6]. In the current study, through in vivo imaging, we examine whether NF-κBp-luciferase expression can be observed in the brains of PRV-infected NF-κBp-luciferase mice. In addition, we evaluated the determinants of PRV-induced inflammatory response that leads to viral encephalitis via the NF-κB pathway.

Lipopolysaccharide (LPS) induction is the most commonly used method to study the activation of the NF-κB pathway. LPS-induced NF-κB activation can in turn activate inflammatory cells (e.g., astrocytes and microglia cells) and can result in neuroinflammation of neuronal cells [7]. The present study uses NF-κBp-luciferase mice to establish a virus-induced encephalitis model for the first time, and evaluates the magnitude of the inflammatory response through the expression levels of COX-2 and iNOS and the activation of the NF-κB pathway. In the second part of this study, we use murine microglia (BV-2) cells to demonstrate a PRV-induced encephalitis that may require the activation of microglia. Our results suggest that PRV infection induces encephalitis via the expression of cytokines, COX-2, and iNOS, which might be initiated through the NF-κB-mediated pathway via microglia activation. 

## 2. Materials and Methods

### 2.1. Amplification of the Virus

The porcine kidney cells (PK15) in Dulbecco’s modified Eagle’s medium (DMEM) were complemented with 10% fetal bovine serum (FBS) (Hyclone, Logan, UT, USA) and 1% antibiotics (100 units/mL of penicillin and 100 mg/mL of streptomycin; Invitrogen, CA, USA). The stock of wild-type (wt) PRV (strain TNL) used in this study was amplified from PK15 cells and the titer was determined by a standard plaque assay in PK15 cells [5,6]. The operation of the virus experiment is based on the approval of the Biosafety Committee in the BSL-2 room of our building. 

### 2.2. Cell Culture

The murine BV2 microglial cells provided by Dr. Dah-Yuu Lu (China Medical University, Taichung, Taiwan) were incubated in RPMI-1640 medium containing 10% FBS, 2 mM L-glutamine, and antibiotics at 37 °C in a humidified incubator contained 5% CO_2_.

### 2.3. Animal Model for Detecting the Activation of the NF-kB Pathway

Female 6-week-old FVB/NJNarl-Tg (NF-κBp-luciferase; NF-κBp-Luc)-11Tsai strain transgenic mice were used in all imaging experiments and mortality experiments [8]. FVB/NJNarl-Tg mice were generated from Dr. Pei-Jane Tsai at the National Laboratory Animal Center, Taiwan. First, the animals were subjected to acclimatization for a period of 1 week in a specific pathogen-free (SPF) room with a temperature controlled at 22 ± 2 °C under a cycle of 12 h of light and 12 h of darkness. After one week of acclimation, twenty-four mice were randomly divided into two groups (MOCK and PRV groups), and were compared with each other within the same tested day (day 1 and day 2), respectively. The animals were inoculated with 10 μL (10^5^ plaque forming units (PFU)) of intranasal PRV virus suspension as the PRV group. Mice sham-inoculated with 1X DMEM served as the MOCK group. In the mentioned process, wt TNL-strain PRV was used as the infection agent. After the indicated periods, the mice were anesthetized using 2.5% isoflurane, before they were intraperitoneally (IP) injected with luciferin (a luciferase substrate, 150 mg/kg; Xenogen, Alameda, CA, USA), and then the whole mouse body images were captured in vivo and quantified using a charge-coupled-device camera (IVIS, Xenogen Corp, Alameda, CA, USA); data (in pixels/s) were recorded using Living Image 2.11 software (Xenogen Corp, Alameda, CA, USA). After imaging, we euthanized the animals using isoflurane upon completion of blood, brain, and TG collection. 

This study was approved by the Chung Shan Medical University Animal Care and Use Committee (IACUC No. 1283) and all investigations were carried out in accordance with the “Guide to the Care and Use of Experimental Animals”. 

### 2.4. Quantification of the Virus from Both Brain and Trigeminal Ganglia (TG) of Infectied Mice by Plaque Assay

Six PRV-infected mice each at two time points—1 and 2 days post-infection (dpi)—were sacrificed after anesthetization. Their brains and TG were dissected, and half of each organ was weighed and maintained in 500 and 200 μL of DMEM supplemented with 2% FBS, respectively. These samples were homogenized using a pestle and subjected to two cycles of flash freeze–thaw. Virus titers in the PK15 cells were evaluated through standard plaque assay.

### 2.5. Histopathological Analysis with H&E Stain

For histologic analysis, the other half of brain tissues were fixed in 10% formaldehyde for 24 h, dehydrated with an ethanol gradient, embedded in paraffin, and the paraffin brain tissue sections (3 μm) were stained with hematoxylin and eosin (H&E) was performed using an HE staining kit (Vector Labs, Burlingame, CA, USA.), according to the manufacturer’s instructions. Each image of the sections was captured using a light microscope (20× and 400× amplification, Nikon, Japan).

### 2.6. Determination of the Expression Levels of TNF-α, IL-6 and IL-1β in a PRV-Infected Mouse Brain or BV-2 Cells by an Enzyme-Linked Immunosorbant Assay (ELISA)

Brains from PRV-infected or not infected (MOCK) mice (*n* = 6 mice/group) at 1 and 2 dpi were quickly harvested, weighed, and homogenized in 500 μL DMEM. According to the manufacturer’s instructions, the concentrations of TNF-α, IL-1β and IL-6 in the supernatant of homogenates were estimated by ELISA (eBioscience, San Diego, CA, USA.). Each of the executed experiments was repeated three times.

### 2.7. Detection of Inflammory Proteins by SDS Protein Gel Electrophoresis and Western Blotting

After dissecting the brains, 50% of the organ was weighed, maintained in 500 μL of DMEM, washed through the use of PBS, and lysed through the application of a lysis buffer and subsequently maintained for 30 min on ice. The lysis buffer constituents were as follows: 50 mM Tris-HCl (pH7.5), 150 mM NaCl, 1% Nonidet P-40, 2 mM ethylenediamine tetraacetic acid, 1 mM NaVO_3_, 10 mM NaF, 1 mM dithiothreitol, 1 mM phenylmethylsulfonyl fluoride, and 25 μg/mL leupeptin. At 4 °C, the cell lysates were subjected to a 15 min process of centrifugation executed at 12,000 rpm; the derived supernatants were kept at −70 °C until use. A Bio-Rad protein assay system was utilized to measure the protein levels. A total of 20 μg of whole-cell lysate proteins were resolved in the loading buffer, and were electrophoresed in sodium dodecyl sulphate (SDS)/polyacrylamide gel. After transfer on a membrane of polyvinylidene difluoride and blocking with 5% non-fat milk solution, the membranes were then incubated at 4 °C overnight with anti-iNOS, NF-κB, COX-2 antibodies (Spring Bioscience, LabVision, Runcorn, UK), phospho-NF-κB and GAPDH, and β-actin (Abcam, Cambridge, MA, USA). To visualize the indicated proteins, secondary antibody conjugated with horseradish peroxidase were used and then explored through chemiluminescent reagent (ECL Plus^TM^ Western Blotting Reagents, Amersham Biosciences, Boston, MA, USA). AlphaImager 2200 served as the platform for quantitating the optical densities of the protein bands. The results of the protein expression were normalized with β-actin or GAPDH, and the relative expression folds of these proteins were compared with the MOCK group.

### 2.8. Statistical Analysis

For all paired experiments, data are presented as the mean ± SD. The student *t*-test was executed to determine the statistical significance (set at *p* < 0.05) of the differences in the parameters between the experimental and the corresponding MOCK groups. 

## 3. Results

### 3.1. Effects of TNF-α, NO and IL-6 Generation in BV-2 Cells Infected with PRV

**Figure 1:**
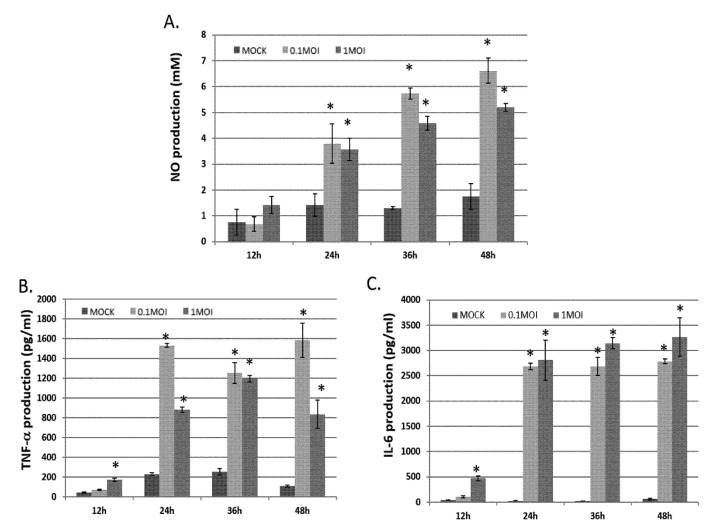
Levels of NO, IL-6 and TNF-α in a mouse brain, either without or with indicated doses of PRV infection. NO, IL-6 and TNF-α production as observed in MOCK (not infected) or infected BV-2 cells of PRV infected 0.1 or 1 MOI for 12–48 h. NO (**A**), IL-6 (**B**) and TNF-α (**C**) concentrations in the culture medium supernatant were determined through ELISA. Data derived are presented as the mean ± SD (*n* = 3). * *p* < 0.05 compared to MOCK group, respectively.

To check whether PRV can trigger pro-inflammatory gene expression in microglia, in vitro, we infected murine BV-2 cells with 0.1 or 1 M.O.I of PRV. Then, assess the levels of NO, IL-6 and TNF-α in the culture medium 12 to 48 h after infection. After 24 h infection, PRV strongly induced NO, IL-6 and TNF-α expression in the BV-2 (Figure 1).

**Figure 2:**
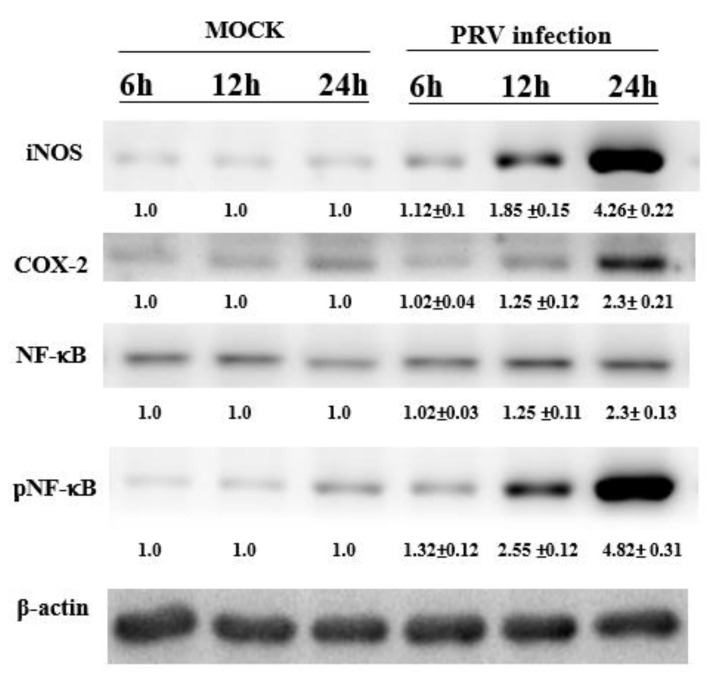
The iNOS, COX-2 and NF-κB p65 expression in non- or PRV-infected BV-2 cells for 12–48 h. Total protein levels in the observed cells were determined through 10% SDS-PAGE and subsequently through Western blotting. The data is normalized with β-actin, and the relative density ratios shown here are the PRV-infected and non-infected groups at the same time point. Data are presented as the mean ± SD (*n* = 3).

To confirm the foregoing results, the iNOS, NF-κB and COX-2 expression levels in the BV-2 cells were determined through Western blotting. The derived results confirm PRV infection to strongly engender iNOS and COX-2 expression in the cells (Figure 2). These findings indicate that in BV-2 cells, the PRV-induced inflammatory response to iNOS as well as COX-2 expression occurs through a pathway that is the activation of NF-κB.

### 3.2. Induction of the NF-κB Signal Pathway In Vivo after the Inoculation of Virus

In this virus-induced mouse model of encephalitis, the activation of the NF-κBp Luc reporter was analyzed by applying the substrate luciferin and detecting luminescence with the in vivo imaging system (IVIS).

**Figure 3:**
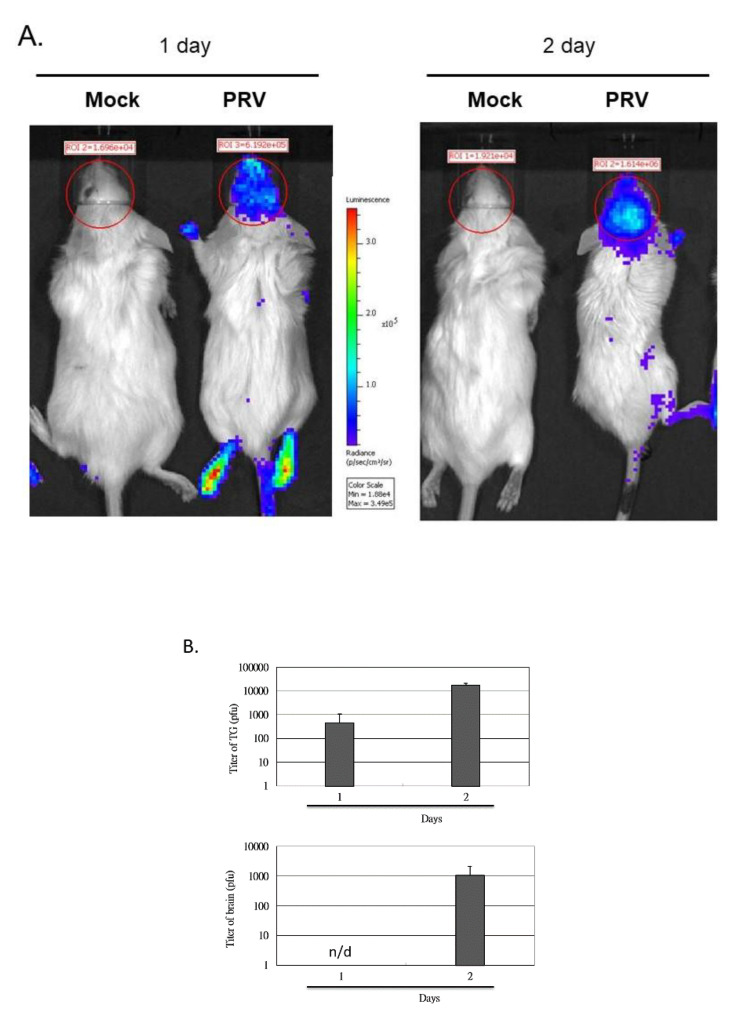
Quantification of the imaging of 6-week-old FVB/NJNarl-Tg (NF-κB-Luc)11Tsai mice after intranasal inoculation of PRV. Activities of NF-κB pathway-triggered luciferase expression were detected in the brain of PRV-infected mice at 1 and 2 dpi (**A**). Replication in the mouse brain and TG. The mice were infected by intranasal inoculation with 10^5^ PFU of PRV and then sacrificed 1 or 2 days later. The mouse TG and brain were excised and processed, and the viral load was determined by a standard plaque measurement (**B**). The data in (**B**) represents the mean ± SD of 6 mice. No virus was detected in the mock group (uninfected). *n*/d = not detected.

We determined if luciferase expression is detectable in infected mouse brains. Figure 3A indicates that the luminescence signals in the brains are significantly detected. The photons/sec/cm^2^/sr within the Regions of Interest (ROI) located in the brain of the MOCK control mouse (one day) are 1.696 × 10^4^, the photons within ROI of the PRV-infected mouse are 6.192 × 10^5^, and the photon ratio of the PRV-infected mice and MOCK mice was more than 36 times higher. In addition, the photon ratio of PRV-infected mice and MOCK mice was more than 80 times higher on the second day.

To understand if the photon levels of ROI correlate with viral infection or replication in brain and trigeminal ganglion (TG), the viral levels in the mouse brains and TG were quantified through in vitro plaque assay (Figure 3B) and compared with the total photons in ROI detected in vivo. 

### 3.3. Histopathological Changes in the Brain Tissues of PRV-Infected Mice

**Figure 4:**
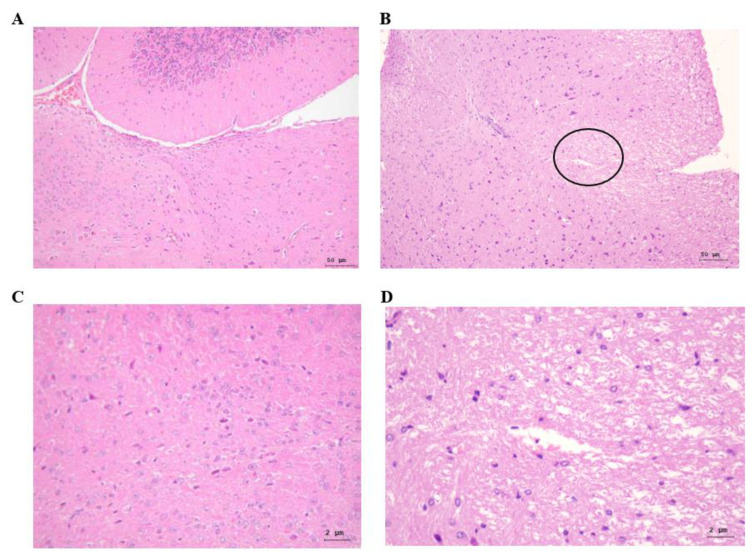
Histopathological changes in the brain tissue of PRV-infected mice. Histopathological findings in the brain of MOCK (**A**,**C**), uninfected and PRV-infected mice (**B**,**D**) at 2 dpi. No significant lesions were found in the brain and stem (**A**,**C**). In the brain stem of PRV-infected mice (**B**,**D**), necrosis (circle) was found. A, B. 20×, C, D. 400×, H/E stain.

To determine the influence of PRV infection on the infiltration of inflammatory cells, 6-week-old NF-κBp-Luc mice at 2 dpi were subjected to imaging, and the brain and TG were excised. Formalin-fixed, paraffin-embedded brain sections were examined through H&E staining (Figure 4). The results showed increased necrosis in the brain of PRV-infected mice at 2 dpi.

### 3.4. PRV-Induced TNF-α, IL-1β and-6 Expression in Mouse Brains

**Figure 5:**
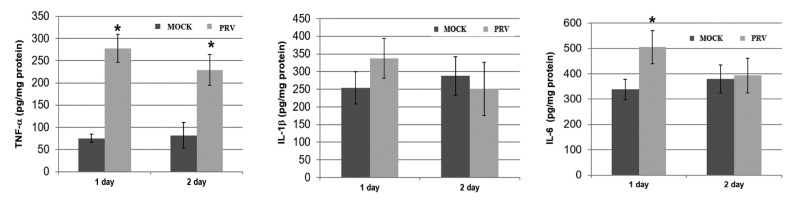
PRV-induced TNF-α, IL-1β and IL-6 expression in the brains of PRV-infected mice. Mice in the uninfected MOCK group and 24 or 48 h after infection with 10^5^ PFU PRV were sacrificed, and the expression levels of these cytokines in the brain were detected by ELISA. Data are expressed as the mean ± SD (*n* = 6). * *p* < 0.05 compared with the MOCK group, respectively.

Previous studies reported that PRV triggers activation-induced inflammation in RAW264.7 cells (macrophage cell line) [5,6,9]. These studies showed that the induction of inflammation in PRV-infected RAW264.7 cells is mediated through the activation of the NF-κB signaling pathway, which is a major transcription factor known to control factors, such as iNOS, IL-1β and -6, COX-2 and TNF-α. Therefore, we evaluated the expression profile of pro-inflammatory cytokines in mouse brains. A significant increase in these pro-inflammatory cytokines was observed in the PRV-infected mouse group (*p* < 0.05; Figure 5). Specifically, the cytokines TNF-α and IL-6 were significantly enhanced, while also increasing IL-1β detected as early as 1 day, and significantly enhanced TNF-α levels remained elevated for up to 2 days. These findings are supported by previous work, which shows that innate immune activation plays an important role in the pathogenesis of viral infections.

### 3.5. Expression of NF-κB, iNOS and COX-2 in a PRV-Infected Mouse Brain

Reactive oxygen and reactive nitrogen species (such as nitric oxide, NO) have a variety of regulating effects on inflammation, and are highly involved in regulating immune responses [10]. iNOS (NOS2) creates sustained and excessive NO, which exerts a proinflammatory and toxic effect. In brain tissues, glial cells as well as neurons express iNOS. Studies reported iNOS up-regulation in inflammatory and infiltrating macrophages, following brain infection by alphaherpes PRV [5,11,12] or HSV type 1 [13]. In addition, COX-2, a major mediator of prostaglandin biosynthesis, was reported to be markedly induced following the herpesvirus (e.g., human cytomegalovirus, HSV and PRV) infection of fibroblasts [5,13]. 

**Figure 6:**
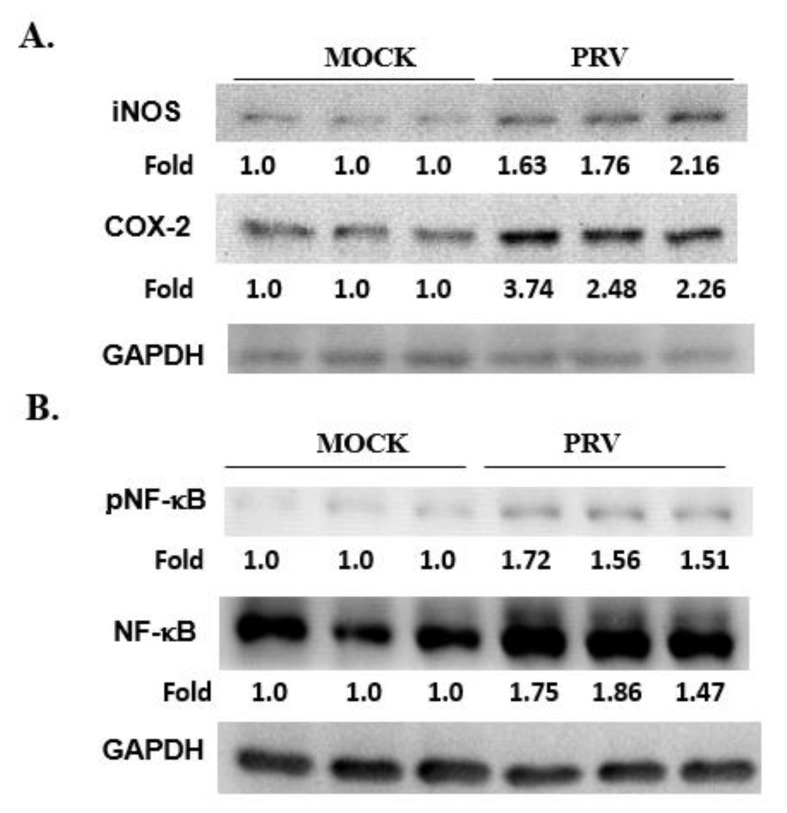
The levels of iNOS, COX-2 and NF-κB expressed in the brains of PRV-infected mice. The expression levels of iNOS, COX-2 and NF-κB in the brains of mice infected with 10^5^ PFU PRV for 48 h or uninfected (simulation group). The total protein level was assessed by 10% SDS-PAGE, followed by Western blotting of iNOS, COX-2 and internal control GAPDH band images. The relative fold change compared with the simulated group samples is shown in (**A**). The levels and relative fold changes of NF-κB, phosphorus-NF-κB (pNF-κB) and GAPDH are shown in (**B**).

In order to determine the relationship between COX-2, iNOS and NF-κB pathways in the inflammatory response to PRV, we examined their expression in the brains of PRV-infected mice. We observed that the expression levels of iNOS, COX-2, NF-κB and phospho-NF-κB (the activated form) increased significantly at 2 dpi (*p* < 0.05; Figure 6), but the first two were not significantly up-regulated at 1 dpi (data not shown). These findings imply that the inflammatory response induced by PRV, including the expression of iNOS and COX-2, occurs after the activation of the NF-κB pathway.

## 4. Discussion

The results showed increased eosinophil infiltration in the brain of PRV-infected mice at 2 dpuronal inflammation is controlled by microglia and leads to increased levels of inflammatory mediators, including TGF-β, IL-1β and IL-6, and TNF-α [14,15,16]. In the brain, the relevant macrophages are microglia that serve as the first line for defense microbe invasion [17,18]. In this study, upon infection, substantial proinflammatory cytokine expression was evident, but exclusively at the later stages (Figure 1). Infected brain tissues exhibited significant IL-6 and TNF-α accumulation, which is also implicated in the neuronal inflammation processes. Following infection, the mediators of the inflammatory cytokines targeted the signaling pathways (e.g., NF-κB) involved in the gene induction of iNOS, and COX-2. LPS-activated NF-κB could trigger neuroinflammation in neuronal cells through the activation of inflammatory cells (e.g., astrocytes and microglia; [7]). Our data evidenced the PRV-induced production of the neuroinflammation response by proinflammatory mediators (IL-6 and TNF-α; Figure 1) via the iNOS, COX-2 and NF-κB pathway (Figure 2) in microglia (BV-2) cells. Therefore, our study is the first to investigate the potential significance of the PRV-induced encephalitis model in mice and its inflammatory response through microglia cells (BV-2; macrophage-cell line). This result is consistent with our previous study indicating that PRV-infected RAW264.7 cells (macrophage-like cell line from a mouse) may induce an inflammatory response through the NF-κB pathway, resulting in NO production and the production of pro-inflammatory chemokines [5,6].

However, its accuracy in characterizing host susceptibility, virus pathogenesis, and severity by different viral strains is still unknown. Studies in our laboratory revealed that RAW264.7 cells produced a strong pro-inflammatory immune response to PRV infection [5,6], indicating that inflammatory stimuli can induce the phosphorylation and degradation of IKK and IκB (which is a cytoplasmic NF-κB inhibitor), and the consequent cytoplasm-to-nucleus translocation of activated p65/p50 (NF-κB subunit) heterodimers. In this study, we evaluated the level of NF-κB-derived luciferase expression in the NF-κBp-luciferase mouse brain after PRV nasal inoculation by in vivo imaging (Figure 3, Figure 5 and Figure 6). In addition, we observed the determinants of PRV-induced inflammation, which leads to viral encephalitis through the NF-κB pathway (Figure 2 and Figure 6). 

Identifying the main determinants of viral disease is crucial to understanding viral pathogenesis. To this end, luciferase can be utilized to track real-time live activity in animals [19]. Due to major technological advances in light detection, bioluminescence imaging has been widely used to characterize tumors [20,21], bacterial infections [22], HSV expression [23] and viral gene expression [24]. For example, in the studies of Luke et al. (2002), KOS HSV-1 recombinant strains expressing luciferase reporter proteins were used, and monitored for infection [23]. However, this can only be used to monitor specific dis-ease sources and lack practicality. 

## 5. Conclusions

From our results, we conclude that NF-κBp-luciferase mice were used to establish a virus-induced encephalitis model for PRV intranasal infection, via iNOS, COX-2 and NF-κB pathways. Therefore, we explored some of the mechanisms involved in the viral-induced encephalitis model. It is expected that the model will be used in the future to monitor the brain inflammation of mice by fluorescent monitoring via IVIS, and to ensure the successful induction of encephalitis in mice by PRV and other viral infections, to avoid the daily sacrifice of mice to reduce the number of mice used. Future research can focus on the inflammatory biomarkers of NF-κB to understand viral encephalitis better, to develop potential targets for new treatment strategies, and to develop drugs for viral inflammatory diseases.

## Data Availability

The data presented in this study are available on request from the corresponding author.

## References

[1] Pomeranz L.E., Reynolds A.E., Hengartner C.J. (2005). Molecular biology of pseudorabies virus: Impact on neurovirology and veterinary medicine. Microbiol. Mol. Biol. Rev..

[2] Roizman B., Knipe D.M., Whitley R. (2007). Herpes Simplex Viruses.

[3] Brittle E.E., Reynolds A.E., Enquist L.W. (2004). Two modes of pseudorabies virus neuroinvasion and lethality in mice. J. Virol..

[4] Brukman A., Enquist L.W. (2006). Pseudorabies virus EP0 protein counteracts an interferon-induced antiviral state in a species-specific manner. J. Virol..

[5] Lin H.W., Chang T.J., Yang D.J., Chen Y.C., Wang M., Chang Y.Y. (2012). Regulation of virus-induced inflammatory response by β-carotene in RAW264.7 cells. Food Chem..

[6] Liu C.W., Lin H.W., Yang D.J., Chen S.Y., Tseng J.K., Chang T.J., Chang Y.Y. (2016). Luteolin inhibits viral-induced inflammatory response in RAW264.7 cells via suppression of STAT1/3 dependent NF-κB and activation of HO-1. Free Radic. Biol. Med..

[7] Stumpf T.H., Shimeld C., Easty D.L., Hill T.J. (2001). Cytokine production in a murine model of recurrent herpetic stromal keratitis. Investig. Ophthalmol. Vis. Sci..

[8] Hung Y.-P., Ko E.-C., Chou P.-H., Chen Y.-H., Lin H.-J., Liu Y.-H., Tsai H.-W., Lee J.-C., Tsai P.-J. (2020). Proton-Pump Inhibitor Exposure Aggravates Clostridium difficile-Associated Colitis: Evidence From a Mouse Model. J. Infect. Dis..

[9] Opdenakker G., Van den Steen P.E., Van Damme J. (2001). Gelatinase B: A tuner and amplifier of immune functions. Trends Immunol..

[10] Gu S.M., Park M.H., Hwang C.J., Song H.S., Lee U.S., Han S.B., Oh K.W., Ham Y.W., Song M.J., Song D.J. (2015). Bee venom ameliorates lipopolysaccharide-induced memory loss by preventing NF-kappaB pathway. J. Neuroinflammation.

[11] Lin H.W., Chen Y.C., Liu C.W., Yang D.J., Chen S.Y., Chang T.J., Chang Y.Y. (2014). Regulation of virus-induced inflammatory response by Dunaliella salina alga extract in macrophages. Food Chem. Toxicol..

[12] Lin H.W., Lee Y.J., Yang D.J., Hsieh M.C., Chen C.C., Hsu W.L., Chang Y.Y., Liu C.W. (2021). Anti-inflammatory effects of Flos Lonicerae Japonicae Water Extract are regulated by the STAT/NF-κB pathway and HO-1 expression in Virus-infected RAW264. 7 cells. Int. J. Med. Sci..

[13] Fujii S., Akaike T., Maeda H. (1999). Role of nitric oxide in pathogenesis of herpes simplex virus encephalitis in rats. Virology.

[14] Guzik T.J., Korbut R., Adamek-Guzik T. (2003). Nitric oxide and superoxide in inflammation and immune regulation. J. Physiol. Pharmacol..

[15] Marcaccini A., López-Peña M., Bermúdez R., Quiroga M.I., Guerrero F.H., Nieto J.M., Alemañ N. (2007). Pseudorabies virus induces a rapid up-regulation of nitric oxide synthases in the nervous system of swine. Vet. Microbiol..

[16] Ray N., Bisher M.E., Enquist L.W. (2004). Cyclooxygenase-1 and -2 are required for production of infectious pseudorabies virus. J. Virol..

[17] Hui B., Zhang L., Zhou Q., Hui L. (2018). Pristimerin Inhibits LPS-Triggered Neurotoxicity in BV-2 Microglia Cells Through Modulating IRAK1/TRAF6/TAK1-Mediated NF-κB and AP-1 Signaling Pathways in Vitro. Neurotox. Res..

[18] DiBona V.L., Zhu W., Shah M.K., Rafalia A., Ben Cheikh H., Crockett D.P., Zhang H. (2019). Loss of Par1b/MARK2 primes microglia during brain development and enhances their sensitivity to injury. J. Neuroinflammation.

[19] Contag P.R., Olomu I.N., Stevenson D.K., Contag C.H. (1998). Bioluminescent indicators in living mammals. Nat. Med..

[20] Contag C.H., Jenkins D., Contag P.R., Negrin R.S. (2000). Use of reporter genes for optical measurements of neoplastic disease in vivo. Neoplasia.

[21] Rehemtulla A., Stegman L.D., Cardozo S.J., Gupta S., Hall D.E., Contag C.H., Rose B.D. (2000). Rapid and quantitative assessment of cancer treatment response using in vivo bioluminescence imaging. Neoplasia.

[22] Siragusa G.R., Nawotka K., Spilman S.D., Contag P.R., Contag C.H. (1999). Real-time monitoring of Escherichia coli O157:H7 adherence to beef carcass surface tissues with a bioluminescent reporter. Appl. Environ. Microbiol..

[23] Luker G.D., Bardill J.P., Prior J.L., Pica C.M., Piwnica-Worms D., Leib D.A. (2002). Noninvasive bioluminescence imaging of herpes simplex virus type 1 infection and therapy in living mice. J. Virol..

[24] Wu J.C., Sundaresan G., Iyer M., Gambhir S.S. (2001). Noninvasive optical imaging of firefly luciferase reporter gene expression in skeletal muscles of living mice. Mol. Ther..

